# Acute care inpatients with long-term delayed-discharge: evidence from a Canadian health region

**DOI:** 10.1186/1472-6963-12-172

**Published:** 2012-06-22

**Authors:** Andrew P Costa, Jeffrey W Poss, Thomas Peirce, John P Hirdes

**Affiliations:** 1 School of Public Health and Health Systems, University of Waterloo, 200 University Avenue West, Waterloo, ON, N2L 3G1, Canada; 2 Hamilton Niagara Haldimand Brant Community Care Access Centre (HNHB CCAC), 310 Limeridge Road West, Hamilton, ON, L9C 2V2, Canada

**Keywords:** Delayed discharge, Alternate level of care, Vulnerable elderly, Length of stay, Acute care, interRAI

## Abstract

**Background:**

Acute hospital discharge delays are a pressing concern for many health care administrators. In Canada, a delayed discharge is defined by the alternate level of care (ALC) construct and has been the target of many provincial health care strategies. Little is known on the patient characteristics that influence acute ALC length of stay. This study examines which characteristics drive acute ALC length of stay for those awaiting nursing home admission.

**Methods:**

Population-level administrative and assessment data were used to examine 17,111 acute hospital admissions designated as alternate level of care (ALC) from a large Canadian health region. Case level hospital records were linked to home care administrative and assessment records to identify and characterize those ALC patients that account for the greatest proportion of acute hospital ALC days.

**Results:**

ALC patients waiting for nursing home admission accounted for 41.5% of acute hospital ALC bed days while only accounting for 8.8% of acute hospital ALC patients. Characteristics that were significantly associated with greater ALC lengths of stay were morbid obesity (27 day mean deviation, 99% CI = ±14.6), psychiatric diagnosis (13 day mean deviation, 99% CI = ±6.2), abusive behaviours (12 day mean deviation, 99% CI = ±10.7), and stroke (7 day mean deviation, 99% CI = ±5.0). Overall, persons with morbid obesity, a psychiatric diagnosis, abusive behaviours, or stroke accounted for 4.3% of all ALC patients and 23% of all acute hospital ALC days between April 1^st^ 2009 and April 1^st^, 2011. ALC patients with the identified characteristics had unique clinical profiles.

**Conclusions:**

A small number of patients with non-medical days waiting for nursing home admission contribute to a substantial proportion of total non-medical days in acute hospitals. Increases in nursing home capacity or changes to existing funding arrangements should target the sub-populations identified in this investigation to maximize effectiveness. Specifically, incentives should be introduced to encourage nursing homes to accept acute patients with the least prospect for community-based living, while acute patients with the greatest prospect for community-based living are discharged to transitional care or directly to community-based care.

## Background

Delays in discharge from acute hospitals are a critical challenge for many health care systems in industrialized nations. These delayed discharges are hospital episodes where a patient exceeds the length of stay deemed medically necessary. They are commonly associated with, though not exclusive to, older adults [1-3]. Delayed discharges represent a minority of hospital cases, yet they often have a substantial influence on patient flow throughout the hospital. This influence includes emergency department crowding (access block), cancelations of day procedures, and poor coordination of sub-acute and community care resources [4,5]. Mounting delays and their influence on overall health system capacity has lead to public pressure and targeted policy activity [1,6-11]. Although delayed discharges have a negative influence on the health care system, they are best understood as a reflection of the underlying mismatch between the needs of patients and their access to appropriate health care services [12-14]. Patients who experience a delayed discharge are at increased risk of accelerated functional decline, social isolation, as well as the loss of independence [15-20]. Above all, delayed discharges are a reflection of health system quality.

A widely accepted, valid, and reliable definition for delayed discharge is lacking. As a result, population-level data are scarce and large investigations are rare. In Canada, acute patients with a delayed discharge are commonly referred to as “alternate level of care” (ALC) patients. The ALC construct is also used to identify delayed discharges in some jurisdictions within the United States [5,21,22]. In Canadian hospitals, an authorized physician or physician delegate designates ALC status when acute care services are no longer medically necessary for the patient [23,24]. A patient that occupies an acute care bed for over a day whilst designated ALC is referred to as an ‘ALC patient’ [25-28].

ALC patients have been the targets of many Canadian provincial health care strategies, and progress has been made to characterize ALC patients using administrative and clinical data [23,26,28-30]. In Canada, the distribution of bed days among ALC patients is positively skewed [28]. This suggests that the majority of ALC patients experience short delays stemming from inefficiencies, while a minority of patients experience long delays due to inadequate resources elsewhere in the health care system. A patient sub-population could be highly associated with the presence of ALC days, but if the magnitude of those days is not excessive then they will not account for a substantial portion of hospital bed inefficiency. Any characteristic (organizational or individual) that is found to influence ALC status needs to be understood in terms of its relationship to total ALC bed days in order to comprehend the effectiveness of any conceivable intervention.

Very few studies have explored long ALC lengths of stay beyond the use of limited administrative characteristics. A Canadian analysis showed that patient demographic factors are not associated with ALC length of stay [29]. Two Canadian studies suggest that patients waiting for nursing home admission (residential care) account for a large portion of ALC bed days [28,31]. Similar results have been found in other jurisdictions [5,32,33]. A comprehensive study that explored Canadian ALC patients waiting for nursing home admission found that some of these patients could be discharged to a community setting with the support of transitional programs and increased community care. The study concluded that no single strategy would likely meet the needs of all ALC patients waiting for nursing home admission [26]. The number of ALC days as well as the proportion of total ALC days need to be examined to understand which patients have the longest stays and account for the largest proportion of total hospital ALC bed days. Research should also establish patient characteristics that are useful for directing interventions and capacity planning [1,34]. The identification of sub-populations with excessive delays would allow for informed strategies.

The objective of this study was to identify and describe ALC patients that account for a substantial proportion of total acute hospital ALC bed days.

## Methods

### Design and setting

A retrospective cohort study was done using all acute hospital discharges occurring from April 1^st^ 2009 to March 31^st^ 2011 within a large health region in southern Ontario, Canada. The health region included over 1.3 million people, accounting for roughly 11% of the provincial population, as well as the largest number of adults over the age of 65 years. This health region contained 12 municipalities with wide variations in population density (rural and urban) and socioeconomic status. The region has 10 hospital corporations – including small community, regional, and large tertiary academic centers - with 2,087 staffed acute care beds. The study determined characteristics of ALC patients that were associated with large proportions of all acute hospital ALC days. Ethics clearance was granted from the University of Waterloo Office of Research Ethics (ORE#16597).

### Data sources

A unique health region-wide business intelligence system containing data from all hospitals and the home care agency was used to access census-level hospital and home care records linked by person level identifiers. Specifically, data from the Canadian Institute for Health Information’s Discharge Abstract Database (DAD) as well as the Hamilton Niagara Haldimand Brant Community Care Access Centre’s Client Health Related Information System (CHRIS) and RAI-Home Care (HC) Assessment System were abstracted.

The DAD contains information on all acute hospital discharges in Ontario, and CHRIS includes information for all home care clients and nursing home admissions in the province. Finally, the region’s RAI HC Assessment System contains RAI HC assessment data for all long-stay home care clients and nursing home admissions. All acute care patients in Ontario waiting for a nursing home admission, and who are not expected to go home, receive the hospital version of the RAI HC assessment to initiate their nursing home application. The RAI HC assessment is a comprehensive assessment of a person’s strengths, preferences, and needs [35,36]. Each assessment generates a set of summary scales, including: the Cognitive Performance Scale (CPS) [37,38], the Depression Rating Scale (DRS) [39-41], and the Changes in Health, End-stage Disease, and Signs and Symptoms (CHESS) Scale [42].

### Sample

The sample included all 17,111 acute hospital admissions designated as alternate level of care (ALC) that were discharged between April 1^st^ 2009 and March 31^st^ 2011. No exclusions were used beyond that of ALC status and discharge date. Case level hospital records were linked to home care administrative and assessment records to determine the presence and status of any nursing home application and RAI HC assessment during the inpatient hospital stay. A patient was identified as waiting for nursing home admission if there was a RAI HC assessment completed during the hospital stay linked to an active nursing home application. If more than one assessment was completed (to reflect a change in patient status) only the most up-to-date assessment was used in order to best reflect patient characteristics at time of discharge. Home care discharge information was used to refine hospital discharge information for patients who were referred to the home care agency for home care service or nursing home application.

### Analysis

Analysis was performed using SAS® Version 9.2 for Windows (SAS Institute, Inc., Cary, NC). Acute care inpatients designated ALC were stratified by nursing home admission status and described using available assessment data. Confidence intervals were employed to assess the generalizability of the study findings to all acute hospital ALC patients. Statistical significance is imputed where confidence intervals do not overlap. All confidence intervals were calculated at the 99% level (alpha = 0.01) and those listed for proportions were based on binomial estimation of the standard error of a proportion.

## Results

Patients designated alternate level of care (ALC) and discharged between April 1^st^ 2009 and April 1^st^ 2011 had a mean age of 77.1 years, where 15.5% were under age 50 and 0.2% were under age 20. The majority of ALC patients (57.7%) were female. ALC patients waiting for nursing home admission between April 1^st^ 2009 and April 1^st^ 2011 had a mean age of 81.2 years, where 7.4% were under the age of 65, 1.1% were under the age of 50, and none were under 30 years old. Here too, the majority (57.6%) were female. The mean time to nursing home (RAI HC) assessment from acute admission was 58.1 days, and the mean absolute time to nursing home (RAI HC) assessment was 39.9 days from the ALC designation. As shown in Table 1, ALC patients waiting for nursing home admission accounted for a substantial portion of all ALC bed days (41.5%) despite accounting for a small proportion of ALC patients (8.8%). ALC patients waiting for nursing home admission had longer acute lengths of stay (mean: 20.8, median: 13 days) and substantially longer ALC length of stay (mean: 82 days, median: 50 days).

**Table 1:** Total acute hospital ALC days among ALC patients waiting for nursing home admission, fiscal 2010-2011

**Sub-Group**	**Proportion of all** **ALC patients**		**Mean Acute** **Length of Stay**	**Mean ALC** **Length of Stay**	**Proportion of** **all ALC days**	
	***% (CI)***	***N***	***Days (CI)***	***Days (CI)***	***% (CI)***	***N***
ALC patients waiting for nursing home placement^*^	8.8 (±0.5)	1488	20.8 (± 0.9)	82.0 (± 2.4)	41.5 (± 0.2)	122090
All other ALC patients (not waiting for nursing home placement)	91.2 (±0.6)	15623	11.7 (± 0.5)	10.5 (± 1.6)	58.5 (± 0.2)	172108

Note: All confidence intervals are 99% (alpha = 0.01) unless otherwise specified.

.Having an accepted nursing home referral as well as a completed RAI Home Care assessment with the nursing home application.

**Figure 1:**
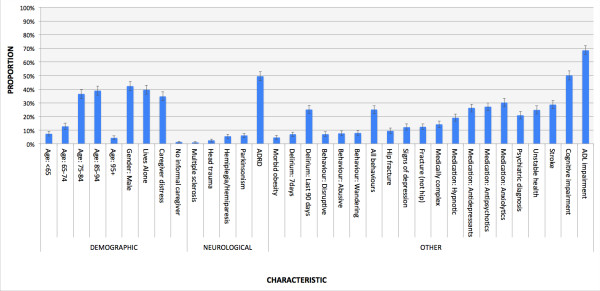
**Characteristics of acute ALC patients waiting for nursing home admission, fiscal 2010-2011.** Note: All confidence intervals are 99% (alpha = 0.01) unless otherwise specified. ADRD = Alzheimer’s Disease and Related Dementias. See Table 2 for elaborations of the included characteristics.

Figure 1 shows that ADL and cognitive impairment were common among ALC patients waiting for nursing home admission – occurring in 50% or more persons. Similarly, the high prevalence of solitary dwelling as well as caregiver distress suggested that many ALC patients waiting for nursing home admission had low informal care capacity. Mood, behavior problems, as well as psychiatric conditions were present in 12% to 25% of persons. The use of psychotropic medication was noted in 65% of ALC patients waiting for nursing home admission. Alzheimer’s disease and related dementias were common, but other neurological conditions were rare. Those with morbid obesity, no informal care, as well as being at the high and low end of the age distribution were relatively rare.

**Figure 2:**
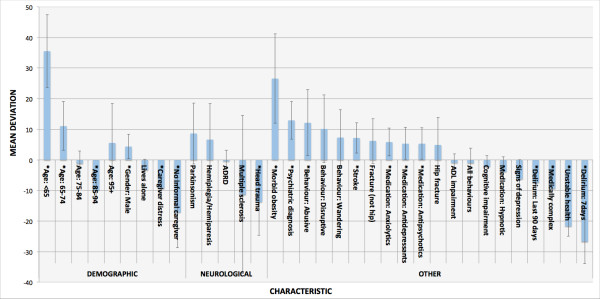
**Mean deviation in ALC length of stay by patient characteristics, ALC patients waiting for nursing home admission, fiscal 2010-2011.** Note: All confidence intervals are 99% (alpha = 0.01) unless otherwise specified. .Significant effect. ADRD = Alzheimer’s Disease and Related Dementias. See Table 2 for elaborations of the included characteristics.

ALC patients waiting for a nursing home admission had a mean ALC length of stay of 82 days. Figure 2 gives the mean deviation (from 82 days) among those ALC patients awaiting nursing home admission, by the characteristics shown in Figure 1. Those less than age 65 years, morbidly obese, with a psychiatric diagnosis, abusive behaviours, aged 65-74 years, stroke, male, as well as receiving of anxiolytics, antidepressants, and antipsychotics had significantly longer ALC lengths of stay. Characteristics significantly associated with shorter ALC lengths of stay were delirium (during last 7 days or 90 days), unstable health, head trauma, having no source of informal care, being aged 85-95, medically complex, as well as caregiver distress. With exception to head trauma, none of the neurological conditions collected were significantly associated with higher or lower ALC length of stay relative to the mean. Many characteristics show very little influence on acute ALC length of stay or have no significant influence.

**Figure 3:**
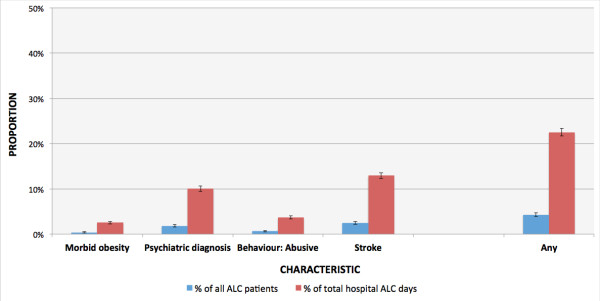
**Proportion of all acute hospital ALC patients and total acute hospital ALC days accounted for by ALC patients waiting for nursing home admission by significant and independent characteristics, fiscal 2010-2011.** Note: All confidence intervals are 99% (alpha = 0.01) unless otherwise specified. See Table 2 for elaborations of the included characteristics. Any = Any of: Morbid obesity, psychiatric diagnosis, abusive behaviours, or stroke.

All characteristics that were significantly associated with ALC length of stay were subjected to a linear regression analysis to identify independent effects. Figure 3 shows the independent groups with significant associations found in Figure 2. Morbid obesity, psychiatric diagnosis, abusive behaviors, and stroke represent near exclusive groups that were highly correlated with demographic characteristics and the provision of psychotropic medication. Overall, persons with morbid obesity, a psychiatric diagnosis, abusive behaviors, or stroke accounted for 4.3% of all patients designated ALC and 23% of total acute hospital ALC days between April 1^st^ 2009 and April 1^st^, 2011.

Table 2 gives the clinical profiles for acute ALC patients with characteristics that were significantly associated with longer ALC lengths of stay. Groups shown in Table 2 were not significantly correlated (also evidenced by a comparison of the proportions) and represent independent effects. ALC patients waiting for nursing home admission that were morbidly obese were younger and more likely to have previously resided in a nursing home. They were more likely to have physical impairments and to be medically complex but less likely to have cognitive conditions. Behaviors and use of psychotropic medication were less common relative to all ALC patients waiting for admission. Those with a stroke diagnosis had characteristics that were very similar to all ALC patients waiting for nursing home admission. ALC patients waiting for nursing home admission with a psychiatric diagnosis were younger and more likely to have received psychotropic medication. Those with abusive behaviours were more likely to be male, have caregiver distress, cognitive/neurological conditions, communication difficulty, other behaviours, as well as more likely to have received antipsychotic and hypnotic medication. All groups had similar discharge destinations, where the majority were discharged to a complex continuing care facility to continue to wait for nursing home admission. Roughly equal proportions were discharged directly to nursing home or to their homes with formal home care supports.

**Table 2:** Profiles of clinical sub-groups with significantly longer ALC stays, ALC patients waiting for nursing home admission, fiscal 2010-2011

	**Morbid Obesity (N = 69)**		**Stroke (N = 426)**		**Psychiatric Diagnosis (N = 311)**		**Abusive Behaviours (N = 115)**	
	**% (CI)**	**N**	**% (CI)**	**N**	**% (CI)**	**N**	**% (CI)**	**N**
**Demographic Characteristics**								
Age (mean)	75.2 (±1.3)	69	80.0 (±0.5)	426	76.3 (±0.7)	311	81.8 (±0.8)	115
Age								
< 65	15.9 (±11.3)	11	9.2 (±3.6)	39	17.4 (±5.5)	54	6.1 (±5.7)	7
65 – 74	21.7 (±12.8)	15	15.0 (±4.5)	64	16.7 (±5.5)	52	13.9 (±8.3)	16
75 – 84	43.5 (±15.4)	30	39.0 (±6.1)	166	39.6 (±7.1)	123	33.0 (±11.3)	38
85 – 94	18.8 (±12.1)	13	34.0 (±5.9)	145	24.1 (±6.2)	75	43.5 (±11.9)	50
≥ 95	0.0 (±0.0)	0	2.8 (±2.1)	12	2.3 (±2.2)	7	3.5 (±3.5)	4
Gender								
Male	31.9 (±1.9)	22	46.9 (±0.9)	200	38.9 (±0.9)	121	58.2 (±1.8)	67
Lived alone	29.0 (±14.1)	20	36.2 (±6.0)	154	38.9 (±7.1)	121	33.0 (±11.3)	38
Lived in								
Private residence	33.3 (±14.6)	23	57.5 (±6.2)	245	46.6 (±7.3)	145	61.7 (±11.7)	71
Private residence (HC^.^)	42.0 (±15.3)	29	29.3 (±5.7)	125	29.9 (±6.7)	93	27.0 (±10.7)	31
Board/assisted/group home	8.7 (±8.7)	6	8.2 (±3.4)	35	13.8 (±5.0)	43	7.0 (±6.1)	8
Residential Care	13.0 (±10.4)	9	3.3 (±2.2)	14	5.8 (±3.4)	18	2.6 (±2.6)	3
Other	2.9 (±2.9)	2	1.6 (±1.6)	7	3.9 (±2.8)	12	1.7 (±1.7)	2
**Primary Caregiver Status**								
No Caregiver	1.5 (±1.5)	1	1.2 (±1.2)	5	2.3 (±2.2)	7	2.6 (±2.6)	3
Caregiver lived with client	56.5 (±15.4)	39	56.6 (±6.2)	241	66.9 (±6.9)	208	58.3 (±11.8)	67
Caregiver is a spouse	29.0 (±14.1)	20	27.5 (±5.6)	117	21.5 (±6.0)	67	34.8 (±11.4)	40
Is a child or child-in-law	49.3 (±15.5)	34	54.7 (±6.2)	233	51.5 (±7.3)	160	44.4 (±11.9)	51
Caregiver distress^1^	30.4 (±14.3)	21	35.0 (±6.0)	149	32.5 (±6.8)	101	56.5 (±11.9)	65
**Clinical Characteristics**								
Cognitive Impairment^2^	31.9 (±14.5)	22	57.0 (±6.2)	243	48.2 (±7.3)	150	81.7 (±9.3)	94
Potential Delirium								
Last 7 days^3^	4.4 (±4.4)	3	5.9 (±2.9)	25	7.1 (±3.7)	22	17.4 (±9.1)	20
Last 90 days^4^	8.7 (±8.7)	6	26.8 (±5.5)	114	24.1 (±6.2)	75	48.7 (±12.0)	56
ADL Impairment^5^	82.6 (±11.8)	57	75.8 (±5.3)	323	67.2 (±6.9)	209	73.9 (±10.5)	85
Communication								
Difficulty making self understood	17.4 (±11.8)	12	44.2 (±6.2)	188	32.8 (±6.9)	102	54.8 (±12.0)	63
Difficulty understanding others	23.2 (±13.1)	16	42.7 (±6.2)	182	35.7 (±7.0)	111	61.7 (±11.7)	71
Behaviours^6^								
Any^7^	14.5 (±10.9)	10	25.6 (±5.4)	109	28.9 (±6.6)	90	100.0 (±0.0)	115
Abusive	2.9 (±2.9)	2	8.2 (±3.4)	35	8.7 (±4.1)	27	100.0 (±0.0)	115
Disruptive	4.4 (±4.4)	3	5.4 (±2.8)	23	9.3 (±4.2)	29	30.4 (±11.1)	35
Wandering	2.9 (±2.9)	2	7.5 (±3.3)	32	5.8 (±3.4)	18	31.3 (±11.1)	36
Psychiatric Diagnosis^8^	27.5 (±13.9)	19	23.9 (±5.3)	102	100.0 (±0.0)	311	23.5 (±10.2)	27
Psychotropic Medications^8^								
Anxiolytics	47.8 (±15.5)	33	33.6 (±5.9)	143	64.3 (±7.0)	200	35.7 (±11.5)	41
Antidepressants	23.2 (±13.1)	16	23.7 (±5.3)	101	37.3 (±7.1)	116	29.6 (±11.0)	34
Antipsychotics	10.1 (±9.4)	7	26.1 (±5.5)	111	40.8 (±7.2)	127	55.7 (±11.9)	64
Hypnotic	15.9 (±11.4)	11	17.1 (±4.7)	73	27.0 (±6.5)	84	28.7 (±10.9)	33
Fractures^**8**^								
Hip	5.8 (±5.8)	4	9.2 (±3.6)	39	10.0 (±4.4)	31	13.0 (±8.1)	15
Other	7.3 (±7.3)	5	11.0 (±3.9)	47	15.4 (±5.3)	48	10.4 (±7.3)	12
Stroke^8^	29.0 (±14.1)	20	100.0 (±0.0)	426	32.8 (±6.9)	102	30.4 (±11.1)	35
Morbid Obesity	100.0 (±0.0)	69	4.7 (±2.6)	20	6.1 (±3.5)	19	1.7 (±1.7)	2
Medically Complex^9^	24.6 (±13.4)	17	12.2 (±4.1)	52	13.2 (±4.9)	41	8.7 (±6.8)	10
Unstable Health^10^	33.3 (±14.6)	23	22.3 (±5.2)	95	21.9 (±6.0)	68	36.5 (±11.6)	42
Neurological Conditions								
ADRD^11^	24.6 (±13.4)	17	46.2 (±6.2)	197	44.4 (±7.3)	138	73.9 (±10.5)	85
Head trauma	1.5 (±1.5)	1	2.8 (±2.1)	12	4.2 (±2.9)	13	7.8 (±6.5)	9
Hemiplegia/Hemiparesis	5.8 (±5.8)	4	15.3 (±4.5)	65	5.5 (±3.3)	17	3.5 (±3.5)	4
Multiple sclerosis	2.9 (±2.9)	2	0.5 (±0.5)	2	1.6 (±1.6)	5	0.0 (±0.0)	0
Parkinsonism	7.3 (±7.3)	5	4.5 (±2.6)	19	7.7 (±3.9)	24	7.0 (±6.1)	8
**Discharge Destination**								
Residential care	15.9 (±11.4)	11	17.4 (±4.7)	74	14.5 (±5.1)	45	20.0 (±9.6)	23
Home (with Home Care Services)	23.2 (±13.2)	16	11.9 (±4.0)	51	13.8 (±5.0)	43	13.9 (±8.3)	16
Transfers								
Complex Care (waiting for placement)	57.9 (±15.3)	40	66.9 (±5.9)	285	65.6 (±6.9)	204	58.2 (±11.9)	67
Other	1.5 (±1.5)	1	0.5 (±0.5)	2	1.3 (±1.3)	4	0.9 (±0.9)	1
Died	1.5 (±1.5)	1	2.8 (±2.1)	12	3.9 (±2.8)	12	5.2 (±5.2)	6
Other	0.0 (±0.0)	0	0.5 (±0.5)	2	1.0 (±1.0)	3	1.7 (±1.7)	2

Note: All confidence intervals are 99% (alpha = 0.01) unless otherwise specified.

^.^ ‘HC’ = Home Care.

^1^ Primary caregiver expresses feelings of distress, anger or depression.

^2^ Based on the interRAI Cognitive Performance Scale (CPS) levels ≥3. Equivalent to 15 - 1 MMSE [37,38].

^3^ Sudden or new onset/change in mental function over last 7 days (from assessment date).

^4^ In last 90 days (from assessment date) person has become agitated/disoriented such that their safety is endangered or requires protection.

^5^ Based on the interRAI ADL Hierarchy Scale levels ≥3 [43].

^6^ Any such occurrence in the last 3 days.

^7^ Any of: Abusive (physically or verbally), Disruptive (incl. Socially inappropriate), wandering, and resisting care.

^8^ Doctor has indicated is present and affects patients status, requires treatment, or symptom management.

^9^ Any receipt of the following in the last 7 days: respiratory treatments, haemodialysis, or tracheotomy care.

^10^ Based on the interRAI Changes in Health, End-stage Disease, and Signs and Symptoms (CHESS) Scale [42], levels ≥3.

^11^ ADRD = Alzheimer’s Disease and Related Dementias.

## Discussion

Acute ALC patients waiting for nursing home admission contribute approximately four times as many inappropriate bed days relative to their proportion. In particular, those with morbid obesity, abusive behaviors as well as a diagnosis of stroke or psychiatric condition account for a greater proportion of total acute hospital ALC days. In Canada, attention should focus on the ALC patient sub-populations identified in this investigation to identify potential solutions for delayed discharges.

The finding that increased non-medical days was associated with waiting for nursing home admission has been reported elsewhere [5,28,31-33]. Other studies have found increased non-medical days among acute inpatients to be associated with stroke [13,23,31,44,45], neurological conditions, and psychiatric disorders [23,46-48]. Also, a U.S. study found that behaviors were predictive of delayed discharges [49], but no investigation has identified the particular behaviors that drive the effect. ALC patients that exhibited abusive behaviors had significantly more ALC bed days relative to the average. Morbid obesity was significantly associated with longer delays among ALC patients waiting for nursing home admission. Obesity has not been explored in previous investigations, likely due to the lack of relevant data in hospital records. Cognitive impairment and informal care capacity were common conditions among all ALC patients waiting for nursing home admission and have been linked to the presence of non-medical days in other investigations [2,23,33,48,50]. Demographic characteristics and particular classes of psychotropic medications were significantly associated with longer ALC lengths as well as highly correlated with the identified clinical subpopulations. Significant increases in ALC length of stay for the identified clinical sub-groups reflect the difficulty in arranging nursing home placement for persons that require unique care equipment and considerable care resources.

ALC patients with signs of potential delirium, informal care distress, head trauma, unstable health, and medical complexity had significantly fewer ALC bed days relative to other ALC patients waiting for admission. ALC patients with potential delirium may have better prospects for nursing home admission if their cognitive status improved. Likewise, those with medical complexity and unstable health may have improved prospects for an immediate nursing home admission if their health status stabilized. The likelihood for improvement of acute medical issues is high, relative to geriatric syndromes, given the skill mix in many acute care settings. The finding that those with caregiver distress were negatively associated with increased ALC bed days could relate an increased likelihood of informal caregivers to accept non-optimal placements as a result of strain and stress. None of these hypotheses could have been addressed in this study and require more investigation.

The province of Ontario has approximately 70,000 publicly funded nursing home beds [51] and approximately 1.6 million citizens over the age of 65 [52]. This investigation suggests that acute care patients with the highest needs experience greater barriers in the nursing home admission process. This finding is similar to the an Australian simulation study that found that the acute hospital sector acts as a safety net for persons who cannot find appropriate placement in a nursing home [53]. The inherent problem in this situation is that those with the most potential for transition to community-based care are those that are admitted to nursing homes, whereas those with the least potential for returning to community-based care remain in the acute hospital. Although a reflexive response to this problem may be to increase supply of nursing home beds, a more careful exploration of other possible alternatives is required. Without alternatives, the demand for nursing home beds will almost certainly continue to be greater than the capacity of that sector. A recent provincial report suggests that nursing home capacity would need to increase by 75% over the next decade to meet the projected demand [30].

A more sustainable strategy to increasing nursing home bed supply will likely involve better management of existing nursing home bed capacity. Specifically, there should be incentives for nursing homes to admit high needs patients from acute care. This change in incentive would require a sustained increase in funding to ensure that nursing home staff have the necessary skill sets, and it may also require facility redesign to sustain quality of life and quality of care. This investigation suggests that the capacity to address weight, neurological, psychological, and behavioral conditions is underrepresented in nursing homes and should be the focus for increasing nursing home capacity. A funding formula that considers resident case mix would be an essential tool for eliminating disincentives to admit acute patients with complex needs. Additional incentives could be considered [54], and some of this may be supported through a reallocation of funding from sectors with cost reductions (i.e., hospitals) to those with increased costs associated with caring for more complex populations (i.e., home care and nursing homes).

Many older adults have negative views of nursing home settings. As such, nursing homes should be considered as the option of last resort rather than a default destination. Community and congregate living arrangements, though preferred, are unlikely living arrangements for many of those with significantly longer non-medical days. However, lighter care acute care patients can be considered for transitional care programs that will allow them to live in a community or congregate living arrangement. Such living options also need to be available for older adults who are discharged from acute hospitals but are no longer able to live independently.

Ultimately, improved resources and coordination are needed in acute hospitals to address the pattern of increasing dependence that then leads to decreasing discharge potential. Evidence suggests that older adults admitted to acute medical units deteriorate as well as acquire new geriatric syndromes [55]. Elder friendly ALC units have been described in the literature [21]. These units have structural changes are designed to increase socialization, improve orientation, and decrease barriers to locomotion. These units also feature staff reorganization where acute nursing is reduced and personal supports as well as allied health professionals are increased. Such units are have been found to reduce staff injury [56,57]. Likewise, the use of rehabilitation has been shown to reduce delayed discharge rates [58]. A U.S. study found that early and continuous discharge planning reduced ALC days [5]. Evidence suggests that discharge planning should commence at admission and reflect the changing status of a patient such that their discharge potential improves as independence is maintained or improved. Hospitals expend valuable staff resources reacting to bed supply crises and may be better served by a focus on early discharge planning [8]. This investigation also identified the need for psychogeriatric services or psychiatric consult services within elder friendly acute units. Case finding tools in the emergency department show promise to stream acute medical admissions into early case management programs or divert directly into post-acute restorative/transitional programs [59].

### Limitations

Given that the assessment data captured in this study did not necessarily reflect the characteristics of ALC patients at time of admission to a nursing home or at ALC designation, the characteristics presented cannot be assumed to be predictive of excessive non-medical lengths of stay. The results of this study do not reflect the characteristics of ALC patients that were not in application to a nursing home. Further, given that this study examined records from a particular health region during a particular time period, the results may not be generalizable to all jurisdictions. Persons comparing these data to data available through other reporting systems should note that data from discharged records do not necessary reflect data from real time reporting systems given that ALC days are accrued on discharge.

## Conclusions

This study suggests that a minority of persons with any non-medical acute care days contribute to a substantial proportion of total non-medical days in acute hospitals. Patients waiting for nursing home admission accounted for a substantial portion of non-medical bed days. Those with morbid obesity, abusive behaviors as well as a diagnosis of stroke or psychiatric condition are particularly important sub-population for capacity planning and improved coordination within the health care system.

## Competing interests

The authors declare that they have no competing interests.

## Authors’ contributions

APC proposed the study, completed the data analysis and prepared the manuscript as well as the revisions of the manuscript. JWP reviewed the analysis, and participated in the revisions of the manuscript. TP and JPH participated in the interpretation of the data and in the discussion of the results. All authors read and approved the final manuscript. APC takes responsibility for the paper.

## Pre-publication history

The pre-publication history for this paper can be accessed here:

http://www.biomedcentral.com/1472-6963/12/172/prepub
